# Medical students: what educational resources are they using?

**DOI:** 10.1186/s12909-019-1462-9

**Published:** 2019-01-25

**Authors:** Lucinda Wynter, Annette Burgess, Eszter Kalman, Jack Edward Heron, Jane Bleasel

**Affiliations:** 1 The University of Sydney School of Medicine – Education Office, Faculty of Medicine and Health, The University of Sydney, Sydney, Australia; 2 Sydney Health Education Research Network (SHERN), The University of Sydney, Sydney, Australia

**Keywords:** Question banks, E-learning, Learning resources

## Abstract

**Background:**

The number of resources available to medical students studying a degree in medicine is growing exponentially. In addition to traditional learning resources such as lectures and textbooks, students are increasingly using e-learning tools like commercially available question banks to supplement their learning. Student preference for learning resources has not been described in detail, and a better understanding of the tools perceived to be useful could provide essential information to medical educators when designing and implementing medical curricula.

**Methods:**

We invited 1083 undergraduate and postgraduate medical students from two major Australian universities to complete an online survey. Questions asked students to indicate the frequency with which they use various types of resources when learning new material or when revising previous content.

**Results:**

Approximately one third (32.3%, *N* = 350) of invited participants completed the survey, and of those who responded, the gender distribution was even with a median age of 25 years. Making written notes and reading textbooks were the most frequently utilized resources for learning new material. Online or downloaded question banks were the most frequently used resource for revision. In addition to the use of traditional learning tools, the majority of students report using a variety of e-learning tools including online teaching videos (92%, *n* = 322) and question banks (90.6%, *n* = 317).

**Conclusion:**

Despite the trend towards e-learning, traditional resources like attendance at face-to-face lectures remain the most popular for learning new material. The increasing use of question banks raises potential issues of poor alignment to medical school curricula. With the advantages of exam technique practice, time efficiency and multiplatform availability, their popularity is likely to continue. Evaluation of existing question banks is required to facilitate appropriate integration into the curricula, with equitable access for all students.

## Background

In the last decade, there has been a rapid expansion of educational resources available for medical students. As well as traditional resources such as lectures, textbooks and tutorials, students are increasingly accessing mobile technology and online tools for learning [1, 2], collectively referred to as e-learning tools. The concept of blended learning, incorporating both e-learning and traditional learning tools, is well established [3]. Currently there is little evidence available to indicate which educational resources medical students prefer to use while completing their degrees.

### Smartphones

Reports of the proportion of students who use smartphones for learning medicine are variable. Between 64 and 98% of students have been reported to own a smartphone [1, 4–7] and the numbers are increasing. In a 2013 cross-sectional study in Birmingham, 87% of students reported using smartphones but only 70% found them useful in aiding their medical education [8]. Another study in Leipzig found that only 32.4% of students were using medical apps on their smartphone [4]. A systematic review of survey articles discussing smartphone apps found only 11 were aimed at medical students, possibly explaining the low uptake of apps for study purposes [9]. Only four years later, a simple search for “medical student app” in the iPhone App store yields a staggering 726 results [10], including diagnostic tools, anatomy handbooks, surgical simulators, lab values, drug references and multiple choice questions covering a range of topics^.^.

### Online resources

Students are also utilizing a multitude of other online resources. A recent survey in Illinois of students in their final two years of medical school found students using online tools including Google docs, Youtube, Twitter, Facebook and Wikipedia for study purposes [11]. In a survey of Welsh medical students, 70% report using ‘Meducation’, an online learning tool consisting of videos, problem based learning cases, tutorials and quizzes [12].

### Question banks

Question banks have also emerged as a popular online learning tool. There are several commercially available question banks for medical students and doctors in training, including: ‘Passmedicine’, ‘PasTest’, ‘OnExamination’, ‘Examdoctor’ and ‘NEJM knowledge +’. Each has between 1500 and 6500 practice questions in the form of single best answer or extended matching questions, accompanied by practice exams, quizzes, images and feedback. Students are able to track their own progress over time and often compare their results with other students. They are used by students studying for the United States Medical Licensing Examination [13], and associated with improved rates of passing the Emergency Medicine certification examinations in the United States [14]. In these contexts, students are using the question banks to revise and practice exam technique for specific examinations. It is also possible that students are using question banks more broadly than this, for example, to learn new information for the first time. Harris and colleagues, generated a question bank of student-written multiple choice questions and made them available online to medical students at Cardiff University [15]. They found a significant uptake, with 600 students using the resource within a three month trial period. It is not currently known, however, what proportion of students use commercial online question banks and for what purpose: revision or learning new information.

### Gender differences

Gender differences in the acceptance and use of e-learning have previously been explored. In comparison to females, males have been reported to be more likely to consider e-learning easy to use, useful and efficient [16]. However, a study of first year medical students in Austria found no significant gender difference in attitudes towards e-learning [17]. A systematic review of gender and learning in surgery found no significant difference in the uptake of e-learning resources [18]. It is important to clarify if there is a gender difference in the uptake of new medical education resources to avoid inadvertent gender bias in medical education.

### Lecture attendance

Reported in-person lecture attendance by medical students is also inconsistent. Some argue that lecture attendance is in decline [19, 20], with many students opting to watch recorded lectures online in their own time. One survey of medical students studying molecular biology found 97.9% wanted to be able to access recorded lectures [21]. At Harvard Medical School, a survey of first and second year students reported that 57.2% of students attend lectures, whilst 29.9% watch them online [19]. Another survey of 190 medical students from New York University reported that 80% of students attend lectures and 20% use mainly online or computer based learning [22]. Lecture attendance may vary between institutions, depending on their importance and frequency within the curriculum, quality of lecturers and the content, whether they are available online or if attendance is compulsory. If students are no longer routinely attending lectures, medical educators may need to shift their focus to newer technologies.

Further clarity in how today’s medical students learn is necessary for three key reasons. Firstly, without knowing which of the multitude of resources are being utilised, medical schools are at risk of falling out of touch with their students’ educational needs. Secondly, to ensure consistent delivery of quality education, the most popular resources need to be identified so that their quality and relevance to university curricula can be evaluated. Finally, so that universities can more effectively allocate resources to develop education tools that students are likely to use. This study sought to assess today’s medical students’ preference for educational resources; to investigate, if this preference shifts when learning new materials, or when revising; and if students’ preference for educational resources are related to their age, previous educational exposure or future career aspirations.

## Methods

### Study context

The study was conducted in June 2015 at the University of Sydney and the University of New South Wales (UNSW). Both universities are located in Sydney, New South Wales, Australia. The University of Sydney offers a four-year graduate entry medical program. The University of New South Wales offers a six-year undergraduate medical program. At the time of the study, participants from both universities were in their final two years of study. Most of their teaching occurred at hospital-based clinical schools, within the clinical environment. Assessment methods at both universities included a combination of clinical and written examinations.

### Participants

All (*n* = 1083) penultimate and final year medical students from The University of Sydney and the University of New South Wales, Australia, were invited to participate in an anonymous online questionnaire hosted on SurveyMonkey.

### Data collection and analysis

Students were invited to participate in the study via an email sent from their respective university administrators in June 2015. Two reminder emails were then sent at two week intervals and the survey was closed after a total of six weeks. No remittance or reward was offered to students for participating in the study.

The survey was designed by the authors. Students were asked to identify, on a seven point Likert scale (never, rarely, occasionally, sometimes, often, mostly or always), to what extent resources were used for learning new skills and knowledge, and revising old skills and knowledge, with a list of ten options, ranging from traditional methods of learning to e-learning platforms and apps. The ten options included:

- making written notes
- attending lectures in person
- reading medical textbooks
- using online or downloaded question banks (single best answers and extended questions)
- attending small group tutorials
- watching online teaching videos
- watching lectures online
- consulting medical literature
- using interactive online materials (not question banks)
- using medical apps (other than question banks)

Demographic information was collected including age, gender, level of previous degree undertaken and future career aspirations.

### Statistical analysis

Survey scales employed ordinal measures of self-reported use of resources (Never = 1, Rarely = 2, Occasionally = 3, Sometimes = 4, Often = 5, Mostly = 6, Always = 7). A Wilcoxon Signed Ranked Test was employed to assess if a difference in preference for a particular resource was indicated between learning new content and revision. A Principal Components Analysis was undertaken to investigate whether clusters exist within students’ preference for resources.

### Ethics approval

The University of Sydney Human Research Ethics Committee approved the study (Project No.: 2015/179).

## Results

### Demographics

In total, 350/1083 (32%) of students completed the survey, with a mean respondent age of 25.6 (Range 18 to 30+ years). The gender distribution was even, with male (*n* = 173, 50%) and female (*n* = 174, 50%) students responding to the survey. Of the respondents, 46.3% of students were in their penultimate year (*n* = 162), 53.7% were in their final year (*n* = 188), 65.2% reported to have completed a previous degree while 34.7% were completing their first degree. Around 1% of respondents reported not owning a smart phone (Yes 98.9%, No 1.1%).

#### General uptake of resources

**Fig. 1:**
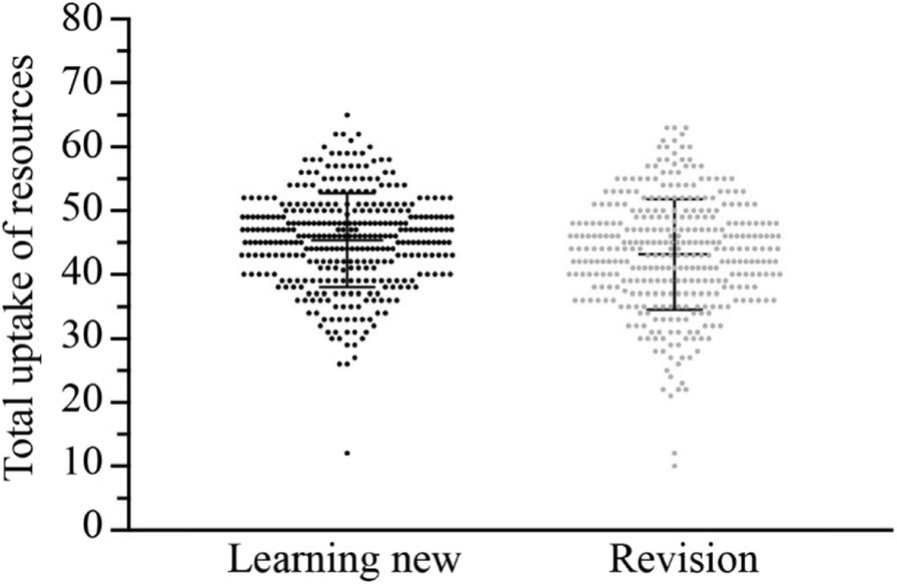
The frequency with which students indicate the use of resources used when learning new materials or revising

Students’ uptake of resources ranged between individuals reporting low use on most items to those reporting high use on most items. The uptake of resources was calculated by converting items of the Likert scale to 0–7 point, where 0 represented “never” and 7 represented “always”. The distribution of responses are summarized in Fig. 1. Frequency was calculated from a conversion of the Likert scale to numerical values. The distribution of the frequency of self-reported resources uptake appears to be similar when learning new content and revising old materials.

**Fig. 2:**
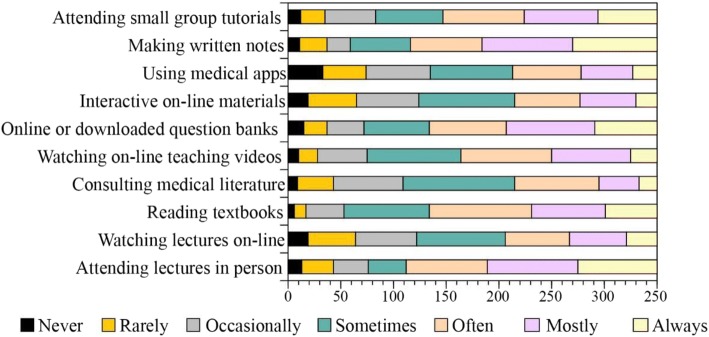
Distribution of terms used by students to indicate the rates of uptake of the common learning resources and tools, when learning new material

Figure 2 shows the distribution of terms used by students to indicate the rates of uptake of the common learning resources and tools, when learning new material.

The learning resources where high-use responses were the most common choice (Always or Mostly) included “Attending lectures in Person” (Mostly 24.5%, Always 21.4%), “Using downloaded online question banks” (Mostly 24%, Always 16.9%) and “Using medical apps” (Mostly 24.6%, Always 22.9%). The most common response did not include any of the low-use (Never or rarely) options. The most common rate of use for the remaining resources were: “watching lectures online” - sometimes (24%); “reading text books” – often (27.7%); consulting medical literature – sometimes (30.2%); watching online teaching videos – sometimes (25.4%); “using interactive online materials” sometimes (26%) and “attending group tutorials” often (22%).

### Resources used when revising old material

**Fig. 3:**
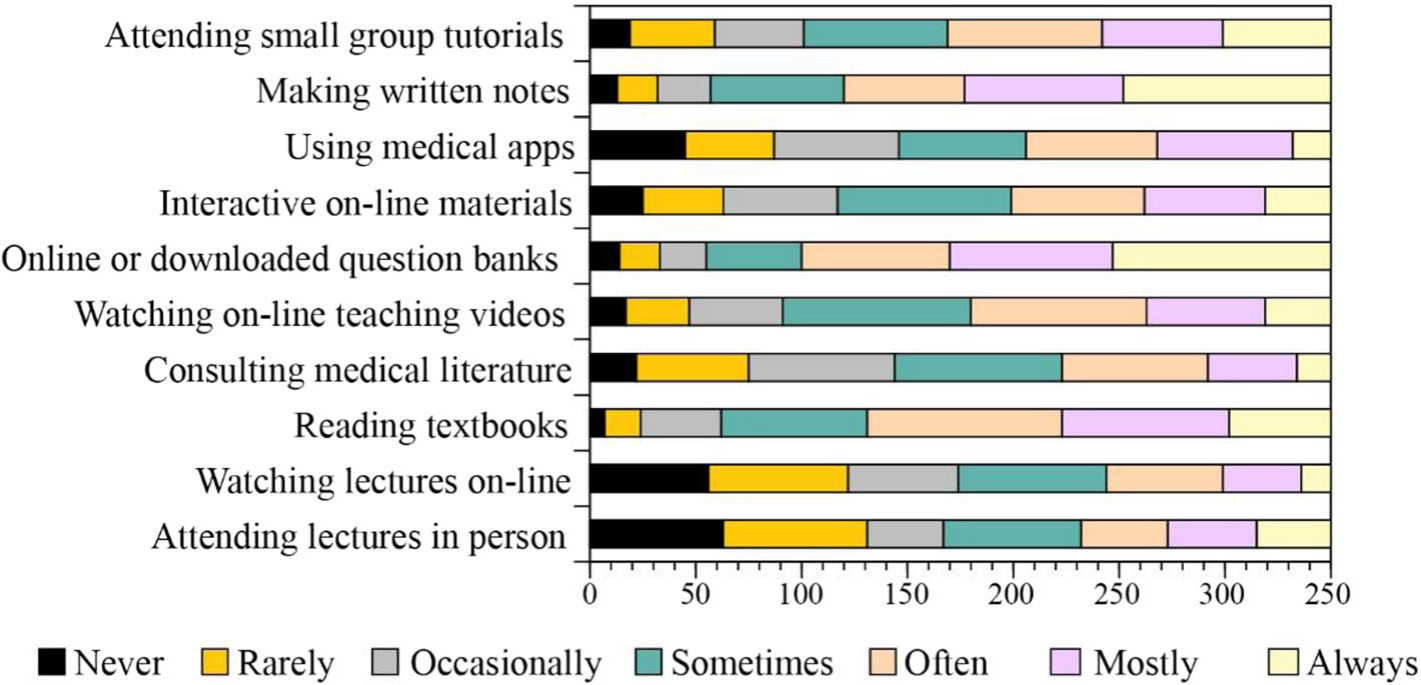
Distribution of terms used by students to indicate the rates of uptake of the common resources and tools, when revising old material

Figure 3 shows the distribution of terms used by students to indicate the rates of uptake of the common resources and tools, when revising old material. The learning resources where high-use responses were the most common choice (Always or Mostly) included “Use of downloadable online question banks” (Mostly 22%, Always 29.4%), “using medical apps” (Mostly 18.3%, Always 5.1%) and “revising written notes” (Mostly 21.4%, Always 28%). The most common response for the low-use (Never or rarely) options was reported only for “attending lectures in person” (Never 18%, Rarely 19.4%). The most common rate of use for the remaining resources were: “watching lectures online” - sometimes (15.7%); “reading text books” – often (26.3%); consulting medical literature – sometimes (22.6%); watching online teaching videos – sometimes (23.4%); “using interactive online materials” sometimes (23%) and “attending group tutorials” sometimes (20.9%).

### Change in preferred resource when learning new and when revising old material

A Wilcoxon Signed rank test revealed a statistically significant difference between the frequencies with which students indicate using the following resources: (i) attending lectures;(ii) watching lectures online; (iii) consulting medical literature; (iv) watching online videos; (v) using online or downloaded question banks; (vi) using interactive online materials and (vii) attending small group tutorials. The effect size and direction of use preference is summarized in Table 1.

**Table 1:** Wilcoxon Signed Rank Test, Comparison of the frequency of use of each educational resource, for either learning new skills and knowledge or revision

	Significance	Z- score	Effect size	Preferred time of use
attending lectures	< 0.0001	−12.215	0.46 (large)	Learning new materials
watching lectures online	< 0.0001	−7.157	0.27 (medium)	Learning new materials
consulting medical literature	< 0.0001	−0.128	0.01 (small)	Learning new materials
watching online videos	0.007	−2.712	0.10 (small)	Learning new materials
using online or downloaded question banks	< 0.0001	−6.190	0.2 (small - medium)	Revision
using interactive online materials	0.022	−2.291	0.08 (small)	Revision
attending small group tutorials	< 0.0001	−3.932	0.15 (small)	Learning new materials

As students use all resources for both revision and learning, a comparison of the frequency of use of each educational resource was undertaken, demonstrating that students attend lectures in person, watch lectures online, attend small group tutorials and consult medical literature significantly more frequently for the purpose of learning new material than they do for revision (see Table 2). Students use question banks and other online interactive materials significantly more frequently for the purpose of revision than they do when learning new material (see Table 2).

**Table 2:** Principal Components Analysis, Oblimin rotation method with Kaiser Normalization

Item	Component 1	Component 2
New – online or downloaded question banks	0.763	
New - interactive online materials	0.734	
Revision – use of interactive online materials	0.729	
New – using medical apps	0.704	
Revision – using online apps	0.701	
Revision - online or downloaded question banks	0.663	
Revision – watching online teaching videos	0.59	
New – watching online teaching videos	0.579	
Revision – watching lectures online	0.392	
New – watching lectures online	0.357	
Revision – attending small group tutorials		0.724
New – attending small group tutorials		0.705
Revision – attending lectures		0.658
New – attending lectures		0.654
New – making written notes		0.477
Revision – using written notes		0.464
Revision – reading textbooks		0.45
Revision – consulting medical literature	0.354	0.431
New – consulting medical literature	0.384	0.389
Age		−0.356
New – reading textbooks		0.333

### Clusters in preference for resource types

A principal components analysis (PCA) was undertaken to assess whether particular preferences for resources showed clustering. Twenty six items were included in the analysis, suitability of the data was confirmed with Kaiser-Meyer-Olkin test (0.664) and Bartlett’s Test of Sphericity (*p* < 0.001). PCA revealed the presence of four components with Eigen Values above the recommended cut off point of 1, however the inspection of the scree plot revealed only two of the factors to be above the clear break point (Catell, 1966). The two retained components account for 30.5% of the variance in the data; component 1 contributing to 17.9% and component 2 to 12.5% of the variance. Oblim rotation technique produced a solution with strong loading by most of the items loading to only one of the components and a correlation effect between the two components (r = 0.57). The clustering into the components indicate that age, gender, career aspirations and completion of a previous degree was not related to the preferred use of learning materials. As illustrated in Table 2, PCA also revealed clustering occurred along the on-line and off-line differentiation of resources with component one containing online resources and component 2 containing off-line resource, please refer Table 2 for detail.

## Discussion

This study sought to explore today’s medical students’ use of educational resources for learning new materials and for revision. The majority of students reported using medical apps, question banks, online interactive resources and online lectures, as well as traditional learning resources. However, their preference for use was dependent upon whether students were seeking to revise or learn new information. Results indicate that the use of question banks is the most popular resource. However, traditional educational formats, including attendance at lectures and tutorials remain the most popular resource for learning new knowledge.

The results indicate that student reporting of the use of resources cluster around whether the resource is online or not. Our results indicate that students likely use online resources similarly, independent of whether it is for revision or leaning of new content. New media and mobile technology has fundamentally changed the way medical students learn new material and consolidate this knowledge. Mobile technology in medical education is now mainstream [23]. This is almost certainly a reflection of the depths to which these new technologies have been incorporated in to our everyday social, emotional and professional lives. Masters et al. explain the uptake of mobile technologies in the context of “learner-centred” educational theory. Learners now engage teaching tools for the specific outcome of becoming a doctor rather than a more teacher-centred approach of previous generations [23]. As a result, students are more self-directed and have greater independence in their learning. Patel et al., reported that resident medical officers perceive mobile learning as an efficient use of their time [24], a result which can likely be extrapolated to medical students. Online resources that can be accessed on multiple platforms are also convenient and provide instant access to information in any setting. For example, using mobile technology, students can look up reference material on a ward round or test their knowledge on the bus on the way home. This facilitates opportunistic learning, which may be of particular importance to those juggling work, family life and study commitments. Recent developments in education technology have seen increasingly more “serious games” being developed for teaching medicine, successfully employing the concept of fun in learning [25]. Online interactive and mobile learning tools may be popular as they are more engaging than traditional teaching tools, with cross-over entertainment appeal to the gaming generation. Online, interactive and mobile learning technologies are easily accessible and convenient for on-the-go, learner-centred medical students and their widespread use is likely to continue to grow.

### Question banks

Our results indicate that question banks have emerged as the most popular revision tool and the most widely used overall e-learning resource. They are used most frequently for revision but also for learning new material. The only other published data on the topic of online questions banks identified a similarly high prevalence of use (82%), an almost universally positive attitude towards online question banks, and a high adoption of a student-developed and university-sponsored online question bank [15]. We hypothesise a number of factors contribute to their popularity, that could be incorporated into novel educational tools. The popularity of question banks for revision may be as a result of the ‘testing effect’. Repeat testing has been shown to improve retention of information as it engages the student in active learning and recall as well as providing a platform for feedback, which improves learning [26, 27]. The immediate feedback also allows students to identify knowledge gaps. Many questions banks also provide high-quality, referenced content explaining why a particular answer is right or wrong. The commercially available question banks provide a score for each section of questions answered, compared with the learner’s previous attempts and with the average scores of their colleagues. This makes answering the questions more like a game with a top score that needs to be beaten. The exposure to high volumes of single best answer questions also allows students to practice exam technique.

The widespread use of question banks by medical students raises two main questions: are they effective and if so, are they available to all students? The majority of question banks are made available on a subscription basis. They have presumably been designed to match students’ perceived needs and to maximise commercial viability. Commercially available question banks have not been thoroughly evaluated in terms of efficacy or quality, and may not align with university curricula or current best practice. Their widespread use suggests that universities should consider formally evaluating popular question banks or develop their own curriculum-focused banks. The possibility of contracting private sector providers with this task is also available. Harris et al. have published their experience developing a framework for a student-authored, clinician reviewed question bank [15]. They achieved a high degree of participation and utilisation in a population of students with a high pre-intervention prevalence of question bank use. With a shift towards commercial resources universities need to take seriously the task of ensuring equitable access to the highest quality and most relevant resources across age, gender, and discipline.

### Lecture attendance

Traditional resources such as attending lectures in person, making written notes and reading textbooks remain, the most utilized resources for learning new material. Although attending lectures or watching them online were identified as the least utilised resource for revision, medical schools should continue to focus on delivering high quality lectures for the purpose of students learning new material. While our results did not identify gender as a determinant of online or off-line resources, others have reported a gender bias in student preference for learning styles. Mehmood et al. studied the personality traits and gender of medical students and found that men were significantly more ‘impulsive sensation seeking’, [28].

### Student age and career aspirations

Students' self-reported age clustered with responses describing the use of off-line resources. This finding may be confounded by the inclusion of a combination of undergraduate and graduate entry medical programs in our study. Unfortunately, our ethics committee approval for this study did not allow for direct comparison between participating medical schools. Students aspiring to a career in ‘general practice’ or ‘other’ were also less likely to attend small group tutorials, which may be a reflection of age, as they tended to be older than their peers.

#### Limitations

The response rate for the study was 32%. It is possible that students who respond to an online survey are more likely to use online learning materials than those who do not. However, both medical schools use online learning management systems and online assignment submission, so it is likely that most students are comfortable with using online platforms.

Inclusion of participants from two different universities potentially weakens this study, as the lecture programs, online resources and assessments will differ between universities which may influence the learning resource preferences of students. Furthermore, the ethics committee approval for this study did not allow for direct comparison between universities and we were therefore not able to draw comparisons between the graduate entry program at the University of Sydney and the undergraduate program at the University of New South Wales.

This was a quantitative study, and use of qualitative data collection and analysis may have provided a deeper understanding of students’ responses.

## Conclusion

This study sought to gain a better understanding of medical students’ preferences for use of existing resources when learning new material, and undertaking revision. Question banks have emerged as a popular learning and revision resource, which are as yet unevaluated by educational institutions. Factor analysis revealed that students describe the use of online resources and the uptake of off-line learning tools in two separate clusters, indicating that the online/offline nature of the material plays an important role in determining their preference for use in both revision and the learning of new materials. An increased understanding of students’ preferences may assist medical educators in design and implementation of medical curricula that is aligned with student needs.

## Abbreviations

UNSW: University of New South Wales
USyd: The University of Sydney
